# Genome characterization of two NADC30-like porcine reproductive and respiratory syndrome viruses in China

**DOI:** 10.1186/s40064-016-3336-5

**Published:** 2016-09-29

**Authors:** Xiangdong Li, Jiajun Wu, Feifei Tan, Yingying Li, Guobiao Ji, Jinshan Zhuang, Xinyan Zhai, Kegong Tian

**Affiliations:** 1 College of Animal Science and Veterinary Medicine, Henan Agricultural University, Zhengzhou, People’s Republic of China; 2 National Research Center for Veterinary Medicine, Cuiwei Road, High-Tech District, Luoyang, 471003 Henan Province People’s Republic of China; 3 OIE Porcine Reproductive and Respiratory Syndrome Reference Laboratory, China Animal Disease Control Center, No. 20 Maizidian Road, Chaoyang District, Beijing, 100125 People’s Republic of China

**Keywords:** PRRSV, NADC30-like, Recombination, HNjz15, HNyc15

## Abstract

**Background:**

The recent emergence of NADC30-like porcine reproductive and respiratory syndrome virus (PRRSV) in vaccinated pigs arose more attentions for the high incidents of mutation and recombination of PRRSVs.

**Findings:**

In this study, we determined full-length genome sequences of two NADC30-like PRRSV isolates from recent PRRSV outbreaks in China. Phylogenetic analysis showed that these two isolates were clustered in an independent branch together with NADC30, an American isolate in 2008. Genetically, HNjz15 shared 95.6 % nucleotide similarity to NADC30 without any exotic gene insertion. By contrast, HNyc15 shared 93.8 % similarity to NADC30 with recombination with VR-2332 and CH-1a. Two more previously reported NADC30-like PRRSVs were also analyzed and had exotic gene insertions with different PRRSV strains in their nonstructural protein genes.

**Conclusions:**

The above results showed the increased mutation and recombination rates of NADC30-like PRRSV under current vaccination pressure and a more pressing situation for the PRRSV eradication and control in China.

**Electronic supplementary material:**

The online version of this article (doi:10.1186/s40064-016-3336-5) contains supplementary material, which is available to authorized users.

## Findings

### Background

Chinese pig industry has been obsessed by porcine reproductive and respiratory syndrome (PRRS) for decades, especially since the outbreak of highly pathogenic PRRS (HP-PRRS) in 2006 (Tian et al. 2007). The causative agent porcine reproductive and respiratory syndrome virus (PRRSV) belongs to the Order *Nidovirales*, family *Arteriviridae* (Conzelmann et al. 1993). PRRSV can be divided into European genotype 1 and North American genotype 2 with VR-2332 and Lelystad as prototypical strains, respectively. PRRSV genome is about 15 kb in length and contains at least 11 open reading frames. ORF1a and ORF1b constitute nearly 75 % of the viral genome and are cleaved into at least 14 nonstructural proteins that are responsible for genome replication and transcription (Zhou et al. 2015b). Three membrane-associated proteins, GP2a, GP3, and GP4 formed a hetero-trimer complex and are involved in virus entry (Li et al. 2016). Three major structural proteins GP5, unglycosyulated membrane protein M, and nucleocapsid protein N locate at 3′ end of genome, and are indispensable for both virion formation and viral infectivity (Hu and Zhang 2014).

Recently, several field isolates of PRRSV had a very unique genetic background and showed the highest nucleotide similarity to a group represented by NADC30, a type 2 PRRSV that has been isolated in Unite States of America in 2008 (Zhao et al. 2015; Brockmeier et al. 2012; Zhou et al. 2015a). These viruses therefore were designated as NADC30-like PRRSV in China. The first two NADC-30 like PRRSVs, HENAN-XINX (access number KF611905) and HENAN-HEB (KJ143621), were isolated in Henan province and their whole genome became available on NCBI in 2013. The clinical symptoms of NADC30-like PRRSV infection were characterized by respiratory disorders of piglets and abortions of pregnant sows in vaccinated pigs which indicate the inability of current commercial PRRSV vaccine to protect NADC30-like PRRSV infection. So far, the disease has been reported to be widely spread in several provinces and led to huge amount of economic losses in China since 2014 (Zhou et al. 2015a).

### Results and discussion

**Fig. 1:**
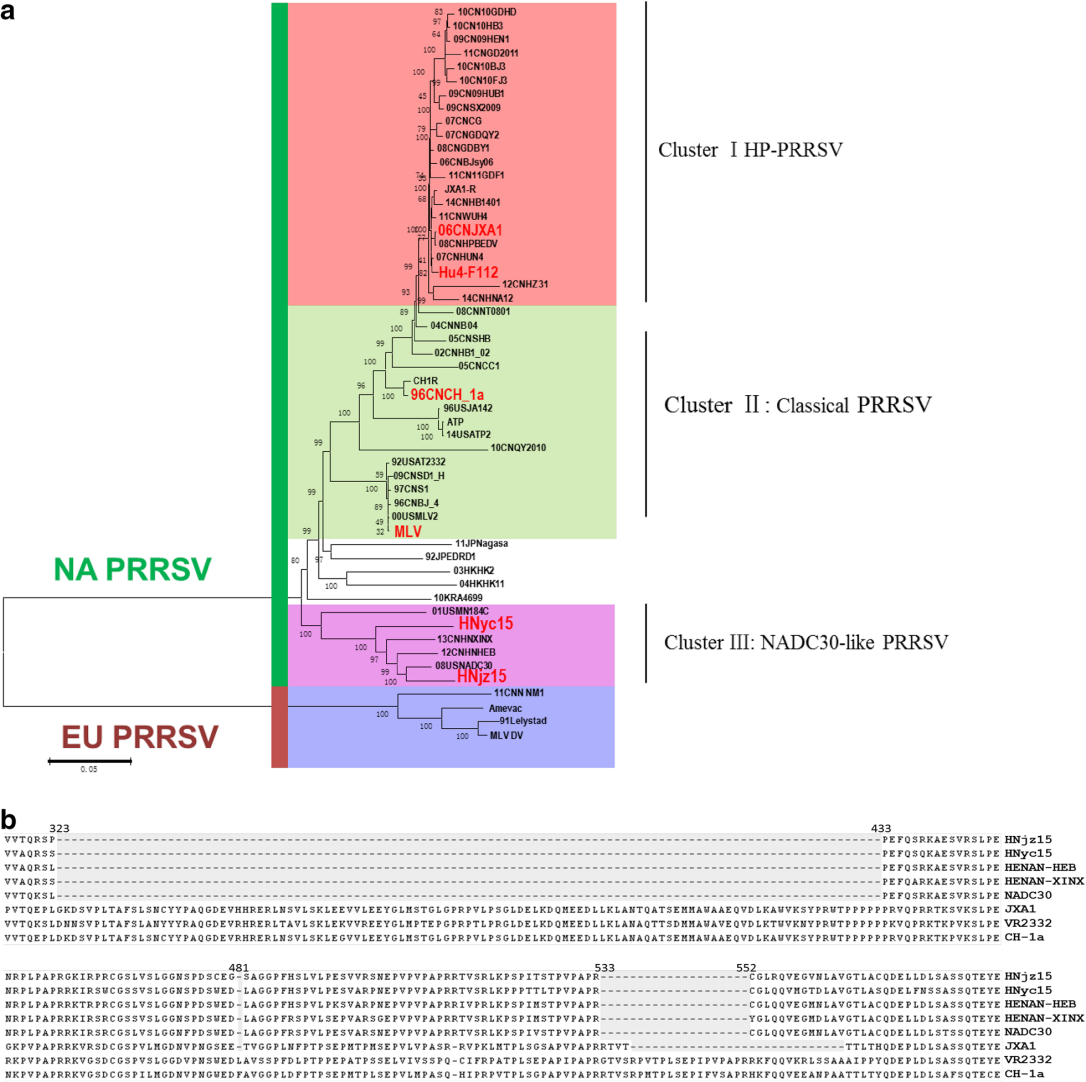
Phylogenetic analysis of whole genomes of HNjz15 and HNyc15 with other 54 PRRSVs (10 vaccine strains and 44 field isolates, virus information are available in the Additional file 1: Appendix) (**a**) and unique discontinuous deletions in nsp2 of NADC30-like PRRSVs (**b**). Each isolates was expressed by isolation years followed by isolation country and name of virus strain. Phylogenetic analysis was performed by using a distance-based neighbor-joining method with 1000 bootstrap replicates in MEGA6. *Numbers along branches* are bootstrap values. *Scale bar* indicates nucleotide substitute per site

In this study, two NADC30-like PRRSVs were isolated from the serum samples of diseased pigs that showed PRRSV clinical symptoms in Henan province. The diseases pigs in this study did not received PRRSV vaccination before. The animal study was approved by the Animal Care and Ethics Committee of China National Research Center for Veterinary Medicine with trial number 2015243, and conventional animal welfare and standards were taken into account. For virus isolation, 50 µl serum of diseased pigs was used to inoculate primary porcine alveolar macrophages (PAM) on a six-well format. After 4 days, the cell culture supernatant was clarified by centrifugation and passaged on PAM and MARC-145 cells. Total RNA was extracted from cell cultures by using an RNeasy Mini kit (Qiagen, Germany) according to the manufacturer’s instructions. The whole genomes were sequenced in triplicates and were assembled with ContigExpress in Vector NTI Advance 11 as described previously (Zhou et al. 2015b). Assembly of the overlapping sequences resulted in complete genomes consisting of 15,019 nucleotides (nt) for both HNjz15 (KT945017) and HNyc15 (KT945018), excluding the 3′ poly (A) tails. A phylogenetic tree based on the full genome sequences of HNjz15, HNyc15, and other 54 published PRRSV strains were generated. Phylogenetic analysis of the whole genome of genome for PRRSV was performed by using a distance-based neighbor-joining method with 1000 bootstrap replicates in MEGA6. As shown in Fig. 1a, together with HENAN-XINX and HENAN-HEB, both HNjz15 and HNyc15 were shown to be genetically more closely related to NADC30 and clustered into a separate branch (cluster III). All HP-PRRSV field isolates and vaccine strains formed another cluster represented by JXA1 and HuN4-F114 respectively (cluster I). Meanwhile, all classical genotype 2 PRRSV vaccine strains and field isolates represented by Ingelvac MLV and Ch-1a were clustered in a separate branch (cluster II).

Genome-wide analysis reveals that HNjz15 and HNyc15 have three discontinuous deletions in the nonstructural protein 2 (nsp2) as previous NADC30-like PRRSVs, which can be used as molecular markers to distinguish them from other PRRSVs (Zhao et al. 2015) (Fig. 1b). The whole genome sequences of HNjz15 and HNyc15 were further compared with NADC30. As shown in Table 1, the nucleotide homology of the complete genome among these three isolates was 93.8–95.6 %. Different gene segments were further analyzed among these three PRRSV strains. Besides nucleotide mutations, there were no gene deletions or insertions for HNjz15 and HNyc15 as compared with NADC30. Both HNjz15 and HNyc15 shared the same length of gene segments with NADC30 except the length of Poly (A) tails. Noticeably, HNjz15 had a mutation (767G-A) that led to a stop codon (766TAG768) and resulted in 255 aa in length in Gp2 protein. The homology of complete genomes of HNjz15 and HNyc15 were also compared with NADC30 on the protein level and the results showed that they shared 97.6 and 98.4 % similarity with NADC30 (Table 1).

**Table 1:** Comparison of complete genomes of HNjz15 and HNyc15 with NADC30 and JXA1

Region	Length				Identities (%)			
	JXA1	NADC30	HNjz15	HNyc15	HNjz15 to JXA1	HNyc15 to JXA1	HNjz15 to NADC30	HNyc15 to NADC30
Nucleotides (bp)								
5′UTR	189	191	190	190	90.5	93.1	97.9	98.4
ORF1a	7422	7166	7166	7166	80.3	79.6	94.9	94.9
ORF1b	4383	4374	4374	4374	87.1	86.4	96.1	95.6
ORF2–7	3188	3188	3188	3188	85.8	87.1	96.0	88.5
3′UTR	150	151	151	151	90.0	88.7	98.0	96.0
Complete	15,347	15,020	15,047	15,047	83.6	83.4	95.6	93.8
Proteins (aa)								
nsp1–8	2473	2372	2372	2372	80.8	80.1	94.1	93.8
nsp2	1166	1065	1065	1065	71.8	70.0	93.2	91.5
nsp9–12	1460	1457	1457	1457	95.8	95.3	98.2	97.8
E	72	73	73	73	90.5	87.8	94.6	89.2
GP2	256	256	255	256	86.4	89.1	96.1	91.1
GP3	254	254	254	254	79.6	83.9	93.7	84.7
GP4	178	178	178	178	86.6	86.6	96.6	88.3
GP5	200	200	200	200	86.6	84.1	96.0	83.6
M	174	174	174	174	93.7	93.7	96.6	96.6
N	123	123	123	123	88.7	91.1	97.6	98.4

An unusual phenomenon of these NADC30-like PRRSVs as compared with other PRRSV variants is the unparalleled incidence of genome-wide recombination with other strains of PRRSV including both classical type 2 PRRSV such as VR-2332 and HP-PRRSV (Zhao et al. 2015). In Zhao’s study, JL580 NADC30-like PRRSV has 6 recombination breakpoints between NADC30 and a HP-PRRSV 09HEN1 (two locate in nsp2, others locate in nps3, nsp7, ORF2a, and ORF4) (Zhao et al. 2015). To explore the recombination of our NADC30-like isolates together with two previously reported two NADC30-like strains (HENAN-XINX and HENAN-HEB), recombination incidences were analyzed by performing similarity within 500-bp window sliding along the genome alignment with 20 bp step size (SimPlot v3.5.1). The analysis results showed that HNjz15 shared 95.6 % nucleotide similarity with NADC30 without any exotic gene insertion, while HNyc15 shared 93.8 % with NADC30 with recombination with VR-2332 and CH-1a between ORF2 and ORF4 (Additional file 1: Appendix Fig. S1A, B). However, it was possible the recombination could occur in cell culture since HNyc15 was propagated on PAM before it was subjected to genome sequencing. By contrast, HENAN-XINX has recombination between NADC30 and VR-2332 in nsp2–5 (Additional file 1: Appendix Fig. S1C), and HENAN-HEB has recombination between NADC30 and JXA1 in nsp2 (Additional file 1: Appendix Fig. S1D). Therefore, unlike the previous PRRSV field isolates, NADC30-like PRRSVs have higher incidences of recombination with both vaccine strains and field isolates.

The recombination of gene segments among different PRRSV strains may lead to the change of virulence (Zhao et al. 2015). In Zhao’ study, the JL580 PRRSV strain was a mosaic NADC30-like virus with HP-PRRSV 09HEN1 recombination at six different sites spanned the genome (Zhao et al. 2015). The pathogenicity of JL580 was tested on 6-week-old pigs and was much higher than that of parental strain NADC-30. One limitation of this study was that we did not perform the animal experiments on non-vaccinated and vaccinated pigs to test the pathogenicity of these two NADC30-like PRRSVs. However, the different recombination patterns of HNjz15 (without recombination), HNyc15 (recombination with VR-2332 and CH-1a), HENAN-HEB (recombination with JXA1), and HENEN-XING (recombination with VR-2332) summarized in this study may provide valuable virus resources to study the change of pathogenicity of PRRSVs through different recombination patterns and genomic sites.

### Conclusion

Two NADC30-like PRRSVs were isolated and analyzed based on the genome level. Phylogenic analysis showed that they are most closed to the NADC30 strain. Genome analysis revealed they undergone unusual frequency of recombination events and became prevalent in China even though the HP-PRRSV still is the dominating strain in Chinese pig herds. The recombination events of these NADC30-like PRRSVs with other PRRSVs are more complex than we expected, and these mosaic PRRSVs show obvious distinct pathogenicity according to the strain they exchanged. The different recombination patterns and pathogenicity of these NADC30-like PRRSVs may lead to more pressing situation for PRRSV control in China.

## Additional file

10.1186/s40064-016-3336-5 Recombination analysis of 4 NADC30-like PRRS strains.
