# Supplementary material for: Genome characterization of two NADC30-like porcine reproductive and respiratory syndrome viruses in China
Source: Springerplus. 2016 Sep 29;5(1):1677. doi: 10.1186/s40064-016-3336-5 (PMC5040649; doi:10.1186/s40064-016-3336-5)
Supplement: Supplementary file 1 — 10.1186/s40064-016-3336-5 Recombination analysis of 4 NADC30-like PRRS strains. [file 40064_2016_3336_MOESM1_ESM.docx]

**Appendix Figure**

**Fig. S1 Recombination analysis of 4 NADC30-like PRRSV strains.** (A) Genome scale similarity comparisons of HNjz15 (query, A), HNyc15 (B), HENAN-HEB (C), and HENAN-XINX (D) with NADC30 (blue), CH-1a (red), JXA1 (green), and VR2332 (yellow). Recombination breakpoints are shown as dark red dotted lines, with the locations indicated at the bottom.

**(A)**

**
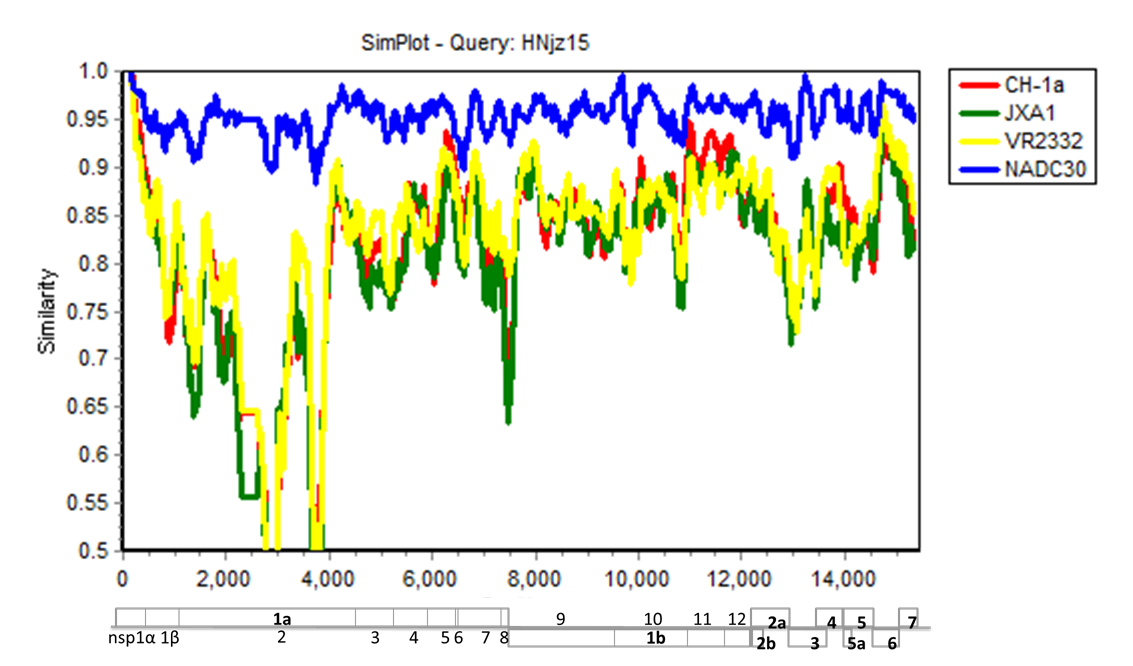
**

**(B)**

**
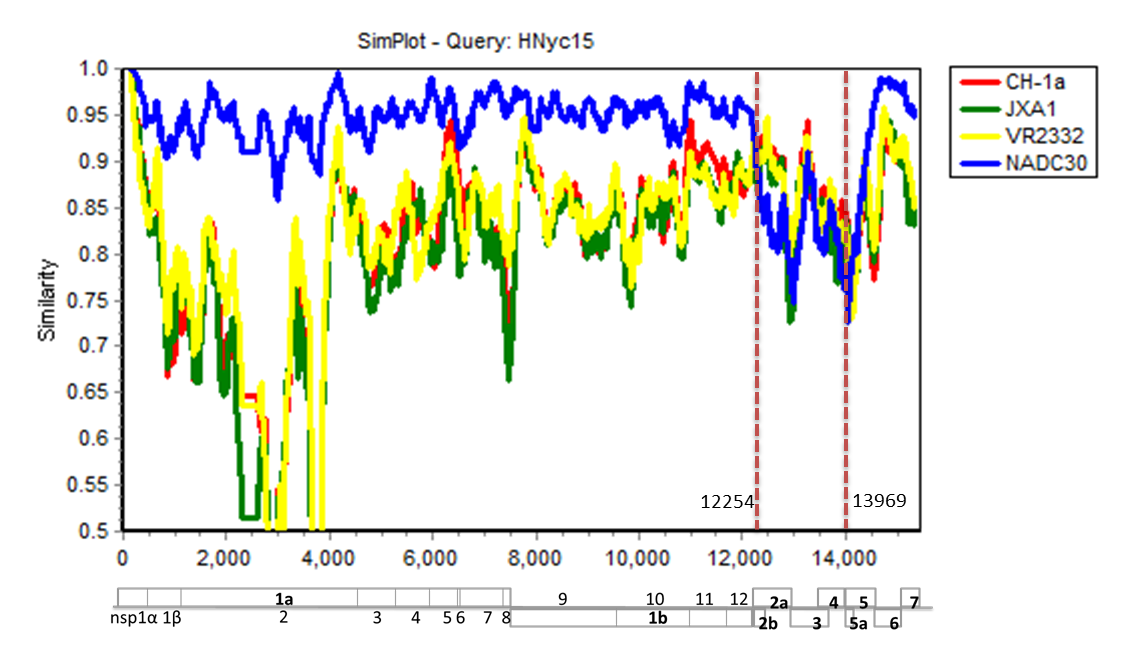
**

**(C)**

**
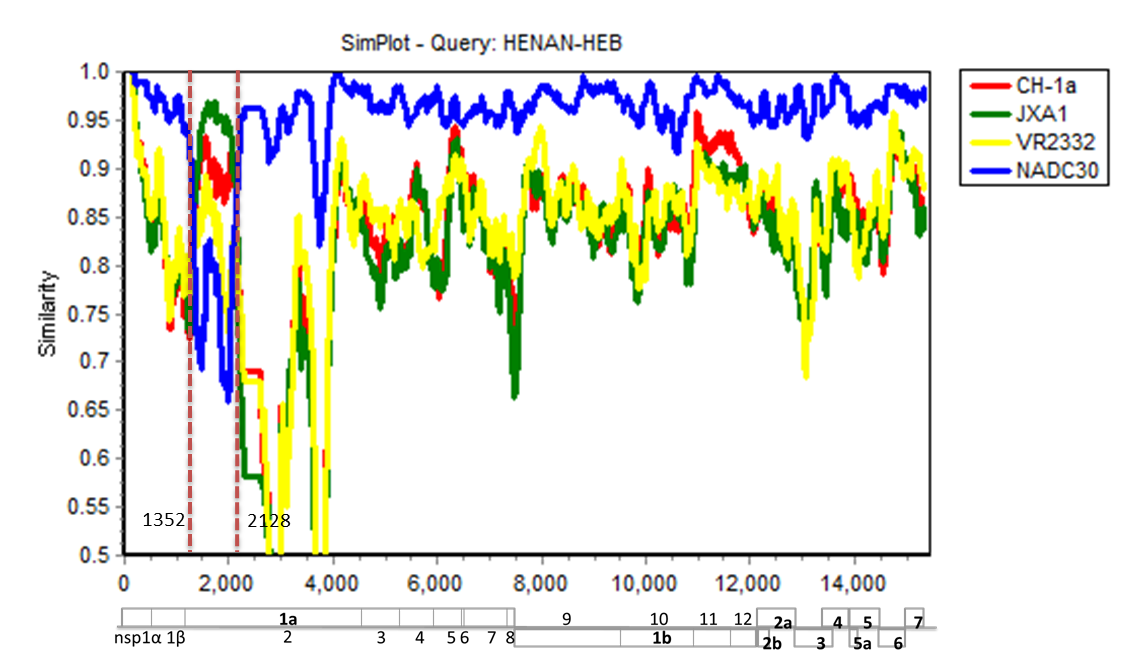
**

**(D)**

**
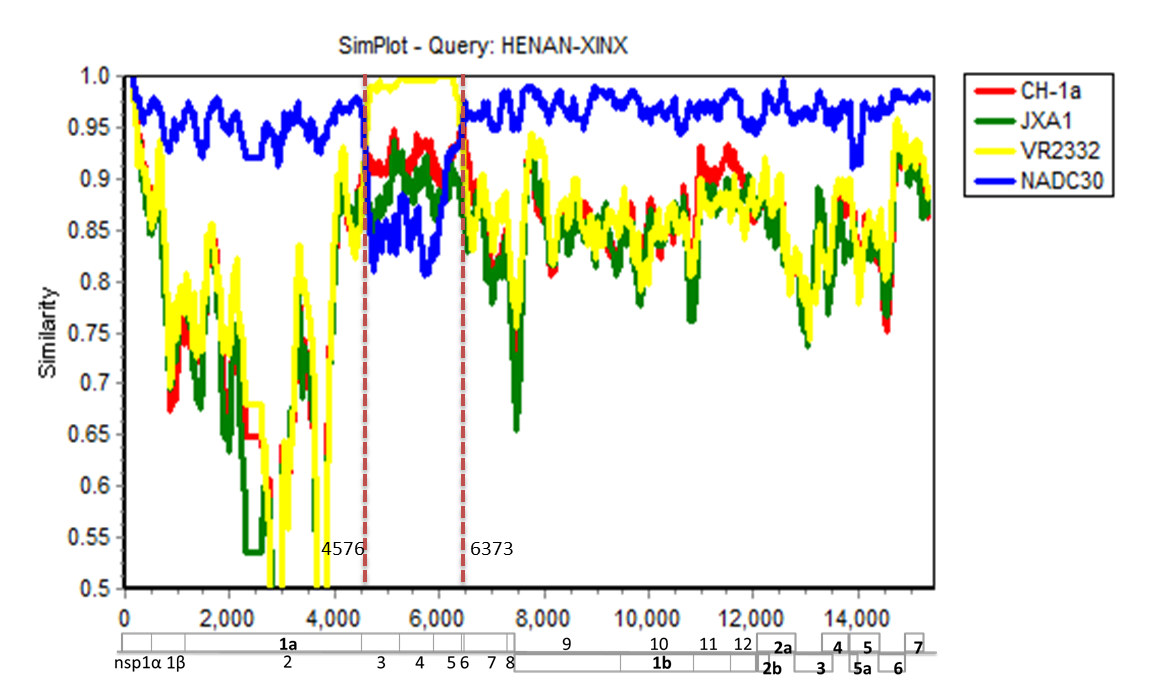
**

| **PRRSV strain** | **Recombinant with** | **Insertion site** |
| --- | --- | --- |
| HNjz15 | \ | \ |
| HNyc15 | VR-2332 and CH-1a | orf2-4 (12254-13969nt) |
| HENAN-HEB | JXA1 | nsp2 (1352-2128nt) |
| HENAN-XINX | VR-2332 | nsp2-5 (4576-6373nt) |

**Table S1. Information of 54 PRRSV strains used in this study.** Vaccine strains were indicated by the names in bold.

| Virus strain | Isolation Year | Country | Access No. |
| --- | --- | --- | --- |
| 10CN10GDHD | 2010 | China | JX215553 |
| 10CN10HB3 | 2010 | China | JQ663553 |
| 09CN09HEN1 | 2009 | China | JF268684 |
| 11CNGD2011 | 2011 | China | KC527830 |
| 10CN10BJ3 | 2010 | China | JQ663542 |
| 10CN10FJ3 | 2010 | China | JQ663548 |
| 09CN09HUB1 | 2009 | China | JF268682 |
| 09CNSX2009 | 2009 | China | FJ895329 |
| 07CNCG | 2007 | China | EU864231 |
| 07CNGDQY2 | 2007 | China | GU454850 |
| 08CNGDBY1 | 2008 | China | GQ374442 |
| 06CNBJsy06 | 2006 | China | EU097707 |
| 11CN11GDF1 | 2011 | China | JX215551 |
| **JXA1-R** | 2009 | China | FJ548853 |
| 14CNHB1401 | 2014 | China | KM261784 |
| 11CNWUH4 | 2011 | China | JQ326271 |
| 06CNJXA1 | 2006 | China | EF112445 |
| 08CNHPBEDV | 2008 | China | EU236259 |
| 07CNHUN4 | 2007 | China | EF635006 |
| **Hun4-F112** | 2010 | China | / |
| 12CNHZ_31 | 2012 | China | KC445138 |
| 14CNHNA12 | 2014 | China | KJ819934 |
| 08CNNT0801 | 2008 | China | HQ315836 |
| 04CNNB_04 | 2004 | China | FJ536165 |
| 05CNSHB | 2005 | China | EU864232 |
| 02CNHB1_02 | 2002 | China | AY150312 |
| 07CNEm2007 | 2007 | China | EU262603 |
| **CH1R** | 2008 | China | EU807840 |
| 96CNCH_1a | 1996 | China | AY032626 |
| 96USJA142 | 1996 | USA | AY424271 |
| **ATP** | 2006 | USA | DQ988080 |
| **14USATP2** | 2014 | USA | EF532801 |
| 10CNQY2010 | 2010 | China | JQ743666 |
| 92USAT2332 | 1992 | USA | U87392 |
| **09CNSD1_H** | 2009 | China | GQ914997 |
| 97CNS1 | 1997 | China | DQ459471 |
| 96CNBJ_4 | 1996 | China | AF331831 |
| **00USMLV2** | 2000 | USA | AF159149 |
| **MLV** | 1998 | USA | AF066183 |
| 11JPNagasa | 2011 | Japan | AB811786 |
| 92JPEDRD1 | 1992 | Japan | AB288356 |
| 03HKHK2 | 2003 | HongKong | KF287133 |
| 04HKHK11 | 2004 | HongKong | KF287138 |
| 10KRA4699 | 2010 | Korea | JX138236 |
| 01USMN184C | 2001 | USA | EF488739 |
| 15HNyc | 2015 | China | KT945018 |
| 13CNHNXINX | 2013 | China | KF611905 |
| 12CNHNHEB | 2012 | China | KJ143621 |
| 08USNADC30 | 2008 | USA | JN654459 |
| 15HNjz | 2015 | China | KT945017 |
| 11CNN_NM1 | 2011 | China | JX187609 |
| **Amevac** | 2009 | Spain | GU067771 |
| 91Lelystad | 1991 | Netherland | A26843 |
| **MLV_DV** | 1999 | Netherland | KJ127878 |
